# The Study of Aviation Safe Incapacitating Device Based on LED Technology with a Smart-Illumination Sensor Unit

**DOI:** 10.3390/s21010081

**Published:** 2020-12-25

**Authors:** Jan Leuchter, Lukas Hon, Radim Bloudicek, Teodor Balaz, Erik Blasch

**Affiliations:** 1 Department of Aviation Technology, Faculty of Military Technology, University of Defence, 66210 Brno, Czech Republic; lukas.hon@unob.cz (L.H.); radim.bloudicek@unob.cz (R.B.); 2 Department of Weapons and Ammunition, Faculty of Military Technology, University of Defence, 66210 Brno, Czech Republic; teodor.balaz@unob.cz; 3 MOVEJ Analytics, Dayton, OH 45433, USA; erik.blasch@gmail.com

**Keywords:** light emitting diodes, LED, dazzler, optical sensors, smart-technology, air safety, health and safety, power electronics

## Abstract

This paper deals with a design and implementation of optical defensive device for protection of aviation personnel. The design is built on the basic characteristics of human eyesight, illumination sensing of the environment, and microcontroller implementation for adaptation over sensed power, flash duration, and person distance. The aviation safe LED-based optical dazzler equipment (ASLODE) utilizes light emitting diode (LED) technology implemented with constant current regulators to control several modes of effects based on situational sensing. The temporarily incapacitating device can be extended by means of real-time illumination sensing to improve power efficiency and reach the highest level of safety. The smart pulse sets the flashing frequency from 8Hz for high-level light intensities and up to 20 Hz in low-level lighting conditions. Experimental results demonstrate the effectiveness of the ASLODE device over numerous experiments with controlled onboard aircraft scenarios that adapt the energy, flash rate, and processing to the sensed environmental illumination to meet aviation hygienic standards for people without eyesight defects.

## 1. Introduction

In aviation, advanced security precautions are strictly required, and modern technologies are able to help to fulfill them. Not only aircraft and aeronautical systems’ requirements are important for air traffic safety, but also equally important is the protection against potentially dangerous behavior of malicious actors. For example, there are precautions for safety monitoring and sensing to minimize the risk of dangerous behavior onboard for passenger protection. In response to a series of severe accidents, a set of hijacking-prevention mandates were approved by commercial airlines as well as aeronautical authorities. One of the group of precautions points at the personnel, trained to use coercive powers e.g., tasers, batons, or pepper sprays. The main aim of these measures is not to cause injury to anyone—the aggressor, who could be a passenger as well as a member of crew; however, should temporarily eliminate or at least reduce his/her attack. The standard level of air traffic safety should be kept, if possible, in such situations. However, in these guidelines, there is no development of adaptive sensing for safe use, such as developing a taser that actively senses the aggressor to determine a controlled level of incapacitating energy output.

There is a possibility of the use of incapacitating defense equipment on board, which is based on the glare principle and following attacker elimination, not to cause injury to him/her or someone else, and/or not to damage any aircraft systems or onboard environment. The paper details an incapacitating defensive equipment design that determines the appropriate level of flash based on the sensed illumination. The design is based on the opportunities to temporarily glare the attacker, safe for the user, protect aircraft systems, and limit the effect on other cabin crew and other passengers. With these safety requirements, the device design is a sensor unit to control power. The primary function is to optically dazzle the aggressor by advanced light flashes based on the sensed distance, without any injuries to the attacker or somebody else’s eyesight in the aircraft. An additional use of such device is the regular torch for normal check of the aircraft cabin. There is the great emphasis on an instinctively and safely usable control design; however, unintended activation must also be taken into consideration as a basic requirement. Additional demands are on batteries and electronics of defensive equipment that follow aviation guidelines and real-time monitoring (e.g., available power level for the electronics).

The article details sensors and microprocessors defensive device settings to operate with the maximum possible efficiency and safe use onboard the aircraft and not to damage aggressor’s, crew’s, or anybody else’s eyesight. A smart-illumination sensor unit based on a light emitting diode (LED) technology scans the basic lighting characteristics of the environment for the Aviation Safe LED-based Optical Dazzler Equipment (ASLODE) performance. From the sensors point of view, the human eye is a complex sensor with high sensitivity and a wide dynamic range capable of perceiving the light intensity in various conditions. The human eye is dynamically able to adapt to lighting conditions to keep visual perception, which requires the ASLODE sensor to coincidentally control the flash response rate and intensity to disable the normal vision, while not damaging the human eye. As for the eyesight damage, the ASLODE response must sense the spectral composition of light (i.e., colors), but the results and analysis in the paper determined that safety can be designed into the device versus active sensing to narrow the spectral content. The use of smart sensors influences the total efficiency, safety, and robustness of the defensive device for use onboard an aircraft in typical lighting conditions.

The paper describes the aviation safe LED-based optical dazzler equipment (ASLODE). Section 2 overviews how the human eye detects and analyzes light. Section 3 details the LED analysis followed by Section 4 of the ASLODE design. Section 5 provides experimental validation. Section 6 presents conclusions.

## 2. Eye-Safe Consideration

In the intended aviation application, there are two competing sensors to be considered: both the human visual sensor (i.e., eye) and the illumination sensor of the device to control the flash. It is noted that smart sensing is required which includes a sensor for the lighting conditions, a sensor to determine the distance to the person, and a sensor to control the power, which combined will be listed together as a smart-illumination sensor unit (SISU). The next section describes in detail the human sensor of the eye.

### 2.1. Human Eye Light Detection

**Figure 1:**
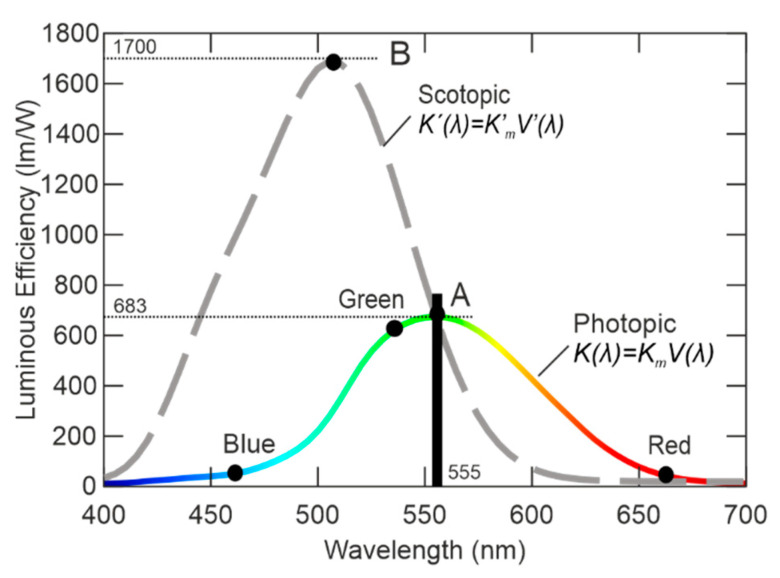
Sensoric characteristic of human eyesight (scotopic and photopic vision) (adapted from [5]).

For the design of incapacitating defensive device, the anatomy and behavior of the human eye are fundamental. The human eye is a complex optical sense organ, perceptive to light and colors containing photoreceptors—rods and cones. Rods are used for the vision in low light and night conditions—scotopic vision. Colors are not recognizable with the scotopic vision. Cones are the eye receptors for full color vision typically in normal light conditions, such as during daytime—photopic vision [1,2,3,4]. There are three types of cones which are sensitive to short, middle, and long wavelength parts of visible light. The human eyesight luminance levels range and types of vision (scotopic, photopic) are shown in Figure 1. Figure 1 shows the curves of luminous efficiency of eyesight in photopic type *K*(*λ*) as well as in scotopic type of vision *K*’(*λ*). Functions *K*(*λ*) and *K*’(*λ*) are based on the relative luminous efficiency *V*(*λ*) for photopic and *V*’(*λ*) for scotopic type of vision. The spectral luminous efficiency function is defined by these equations [5]:

(1) $$K(\lambda )=K_{m}\cdot V(\lambda )\;,$$

(2) $$K^{\prime }(\lambda )={K^{\prime }}_{m}\cdot V^{\prime }(\lambda ),$$

(3) $$K_{555\;\mathrm{nm}}={K^{\prime }}_{555\;\mathrm{nm}}=683\;\mathrm{lm}/W,$$

where *K_m_*= 638 lm/W is a maximum spectral luminous efficiency by 555 nm of wavelength for photopic vision and *K’_m_*=1700 lm/W is a maximum spectral luminous efficiency by 507 nm of wavelength for scotopic vision.

In photometry, the luminous flux is the quantity for light power and it is given by Equations (4) and (5). The luminous flux meets the sensitivity of human eyesight by every part of visible spectrum. The luminous flux is expressed by equations:

(4) $$\Phi_{V}=K_{m}\int_{\lambda }\Phi_{e,\lambda }\cdot V(\lambda )\cdot d\gamma ,$$

(5) $$\Phi_{V}^{\prime }=K_{m}^{\prime }\int_{\gamma }\Phi_{e,\lambda }\cdot V^{\prime }(\lambda )\cdot d\gamma ,$$

where Φ*_e,λ_* [W∙m^−1^] is the spectral radiant flux [5,6].

In Figure 1, there are marked points of maximum sensitivity of human eyesight by photopic vision (at point **A**) and by scotopic vision (at point **B**). The wavelength (*λ*) of point **A** is 555 nm and point **B** is 507 nm. From the luminance efficiency, human eyesight is the most sensitive to green color in scotopic vision as well as in photopic vision. There are also the middle wavelengths of basic colors (Red, Green, and Blue) in the graph of Figure 1.

The conversion for monochromatic light is based on the Candela definition: 1 cd = 1/683 W/sr. From this light conversion, *K*_m_ = 683 lm/W is for the photopic type of vision and the *K*’_m_ value is determined by 1700 lm/W, in order to equal wavelength 555 nm for both types of vision [6].

The function of relative luminous efficiency *V*(*λ*), which is standardized according to CIE (Commission Internationale de I’Eclairage) standards, describes the subjective evaluation of monochromatic brightness to the reference light ratio. An observer sees the reference source of monochromatic light with 555 nm wavelength, where the human eyesight is the most sensitive in photopic vision, and compares it to the brightness of second monochromatic light with other wavelengths. The brightness of reference 555 nm light source is decreased till the observer considers both light sources’ brightness as the same. All visible wavelengths are evaluated in the same way. As for scotopic vision, the human eyesight is the most sensitive to the 507 nm wavelength; therefore, the wavelength of reference monochromatic light source is determined by wavelength 507 nm. In the past, *V*(*λ*) was called the CIE 1931 *V*(*λ*) function, but currently, a modified *V*(*λ*) function is used, which is defined as the CIE 1978 *V*(*λ*) function. The reason of the modification of original CIE 1931 *V*(*λ*) function is the extension of the human eyesight model to include the blue and ultraviolet (UV) parts of the visible part of the spectrum. The CIE 1978 *V*(*λ*) standard most accurately describes the behavior of human eyesight [7,8].

### 2.2. Human Eyesight as an Adaptive Sensor

It is necessary to analyze and utilize all current knowledge about the human eye behavior for the aviation light sensing application, found by minimum flashing as a method for brightness comparison and for *V*(*λ*) determination. Using the glare principle based on the round shaped, light emitted stimulus, a switching frequency of 15 Hz is alternately stimulated by standardized and observed colors. Frequencies lower than 15 Hz color change causes the fusion of colors; nevertheless, if the switching frequency is higher than 15 Hz and the brightness of colors is different, then the flashing is visible [9,10].

Furthermore, it is necessary to take in consideration the ability of eyesight to adapt to light intensity. The human eyesight can mostly perceive the light luminance from 3 × 10^−7^ to 3 × 10^5^ cd/m^2^. Therefore, as to perception in such enormous luminous range, the human eye must be able to set the size of the perception area of the retina. The average rate of diameter adjustment of the pupil is about 400 milliseconds. When the observed events are very fast, the accommodation response of eye is also faster, circa 100 ms [11,12,13].

**Figure 2:**
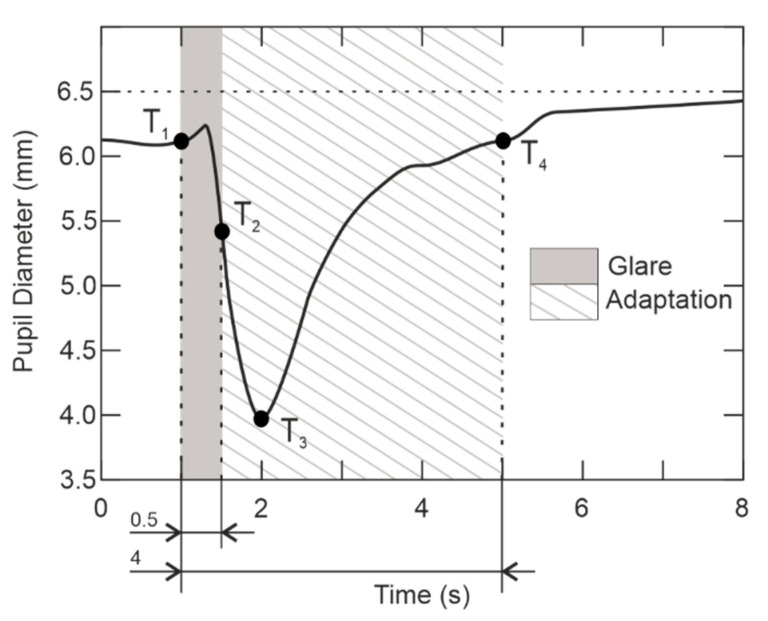
Accommodation response of human eyesight (adapted from [15]).

An experiment [14] verified the rate of response of the saccadic ocular system and the motor system of the hand to a stimulus. The results determined that the speed of saccadic motion reaction is important for our concept and the design of defensive equipment. During the first experiment, the subject follows a fixed point and moves the eye based on the stimulus. The second experiment consists in the connection of motor systems, from which the conditions of the experiment are the same; however, during the stimulus, the subject must move the eye towards the stimulus, and at the same time, press the button. We verified this experiment and the average values of the reaction times are similar. As can be seen from the experiment and its verification in Figure 2, the reaction time of the eye is average 222 ms in the first experiment and 261 ms in the second experiment. The results of the experiment also show that the saccadic movement of the eye is dependent on many factors, including the reaction time of the brain [5,11,14].

In Figure 2, there is the accommodation response of ocular motor system. The curve completely characterizes the behavior of the attacker in our application, and his/her response to glare. It is obvious from Figure 2 that the reaction time of eyesight depends on the ocular motor system, the trigeminal and oculomotor nerves, and also the response of the visual association cortex of the brain [15]. The pupil accommodation is described by curve and T_1_–T_4_ points, where T_1_ is the beginning of glare when the eye tries to adapt the diameter of the pupil and thereby reduce the perceived intensity. At the T_2_-point, the exposure is terminated, however the human eye, by the influence of inertia, continues in reduction of the pupil until T_3_, wherein the pupil begins the extension to the diameter corresponding to surrounding illumination until T_4_ point.

Another problem to consider for the glare device is the dynamic behavior analysis of human eyesight due to light pulse-intensity exposure, light oversaturation, and slower return to default state. Dazzling the eyesight experiment took place in a dark room, where the eyesight was adapted to darkness. Analyzing the peripheral vision in Figure 2, when the observer is temporarily dazzled by a light source with glare illuminance of 15 lx and 60 lx(measured at the place of an observer for a stimulus luminance of 0.5 cd/m^2^), the pupillary response is of 500 milliseconds. Therefore, it was found the eye response is about 300 milliseconds. It follows the previous result and corresponds to estimated response. Figure 2 also shows the fact that the process of return before glare takes almost 4 s. Through the 4s, the illuminance of foveal vision is limited and consequently, the brightness of the observed area is lowered [15].

As shown in Figure 2, the dazzled eye recovers very slowly and the activity of the visual system is disrupted during this process. Permanent repercussion or other injuries of eyesight do not occur with a light load as required for aviation standards. The glare caused by the dazzle device starts as soon as the retina is exposed to higher illuminance and then is adapted, guaranteeing the effective use of the defensive equipment.

For the ASLODE design, it is necessary to design the defensive device, equipped with smart-illumination sensor, not to enable the aggressor’s eyesight fast accommodation to acting light. The human eye can be determined as a sensoric system with limited reaction time depending on the wavelengths of the perceived light and mode of lighting (constant or flashing lighting). With the high luminous efficacy, based on the sensed or operational conditions, the dazzle effect can be adapted in all lighting conditions, which can be onboard the aircraft though the flight. The design of defensive device can utilize such characteristics of eyesight to suppress the sensoric abilities with the step change of lighting parameters that the eyesight cannot accommodate. Variables of the lighting conditions are the spectral composition, lighting mode, and also the amount of emitted light.

For the ASLODE design, it is necessary to take in consideration the various light parameters that influence human eye anatomy. For low-level of luminance, the pupil is more opened to enable more light to get onto retina surface. A sudden change in light characteristics, including the amount of photon, as well as inappropriate spectral composition of light, can damage the retina surface thermally or photochemically, as it is shown in Section 5.3 in detail. The spectral response of ASLODE is based on light emitting diode (LED) technology.

## 3. LED Analysis

For the test case, the influence of attacker eyesight is required such that the light sources act in the visual part of spectrum, with a high level of luminous flux. The high intensity light source with dynamics faster than 100 milliseconds (i.e., 10 Hz flashing) is required. Hence, 10 Hz causes the inability of the attacker’s eyesight to adapt to the light level and the effect will be more intensive. It is also necessary to pay attention to the color of light, affecting the visual system. The light emitted from the source depends on the temperature as well as the perceptive capacity of the observer [10,11,12,13,14,15,16].

Various light sources convert electricity into light. Light sources are generally based on incandescence, luminescence, or quantum generators. Due to the response time, luminous flux, and light efficiency requirements, it is best to use laser or LED (light emitting diodes) as the light source. However, the laser is not suitable for human safety protection.

Currently, the most used technology for light sources is LED technology. LEDs allow emitting the high efficiency light in full color spectrum. The basic element of LED is a semiconductor material with additive elements influencing the emitted color; except if the amount of added element affects the width of forbidden energy gap and related losses. LEDs are semiconductor components based on P-N (positive-negative) junction. The main part of LED is a semiconductor plate type PN, that can emit photons from the active area. Selected semiconductor material determines the wavelength of emitted light. The material also influences the forbidden energy gap and consequently its current-voltage characteristics [5,17,18].

Red colored LEDs use AlGaAs, GaAsP, AlGaInP, or GaP materials [1,2]. The forward voltage of red LEDs is in interval 1.63–2.03 Volts. GaP, AlGaInP, AlGaP, InGaN nebo GaN materials are used for green LEDs and these LEDs have forward voltage from 1.9 to 4.0 Volts according to the material and LED characteristics. Blue shining LEDs are made of ZnSe, InGaN, SiC, or Si additive materials and their forwarding voltage is in the interval of 2.48 to 3.70 V. Only the white color cannot be simply emitted with LED P-N junction with materials addition. There are two possibilities to generate the white color of light. The first one, LED P-N junction generated blue light and it is afterwards transformed in the yellow phosphor or LED is designed as a ultraviolet (UV and the radiated light is let to red, blue, and green colored phosphors. The second possibility uses an LED containing three light emitted chips (red, blue, and green) and white light is the mixture of RGB (Red, Green, Blue) components [19,20].

**Figure 3:**
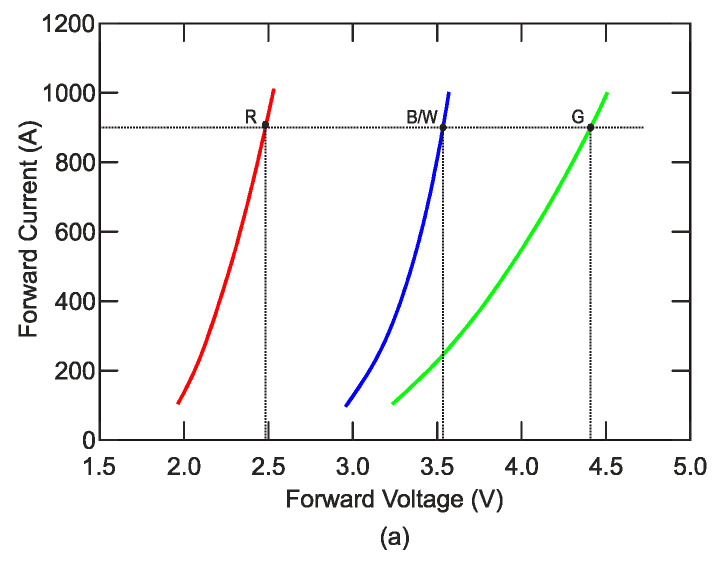

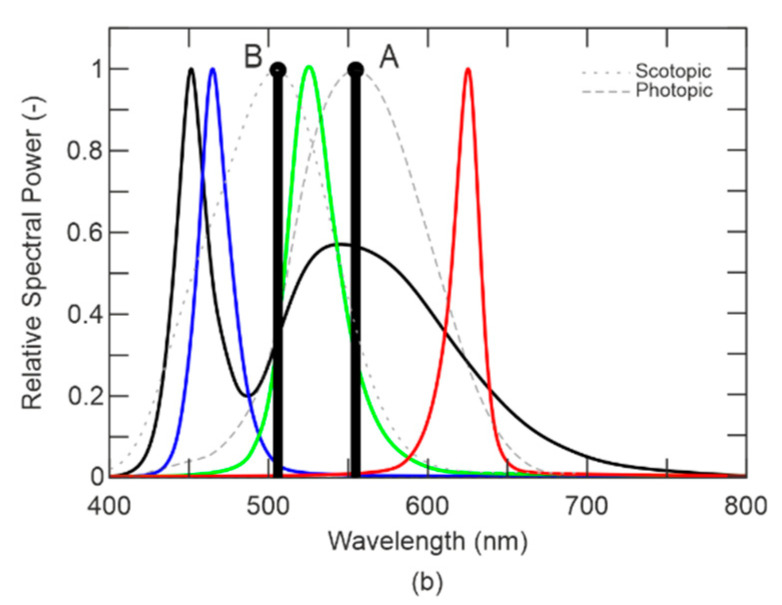
(**a**) RGB current-voltage characteristics; (**b**) typical relative spectral power vs. wavelength (adapted from [21]).

Figure 3 illustrates the typical current-voltage characteristics of red, blue/white, and green LED diodes. Figure 3 also shows the difference in energy of the forbidden gap, where the voltage needed for overcoming of the forbidden energy gap is lesser for the red color than the overcoming voltage for green color. Furthermore, a green LED has the higher forward voltage and less steep characteristics in the forward direction than a red LED.

According to the parameters, a RGBW (Red, Green, Blue, White) LED type LZ4-00MD00 is appropriate for the realization. There current–voltage characteristics are defined by manufacturer in Figure 3a. It is evident that every LED chip has its own chemical composition and consequently its own forbidden gap and power consumption. In case of operating the point setting to nominal current of 900 mA, the LEDs voltage are 2.4V for red, 4.4 V for green, 3.5 V for blue, and 3.5 V for white. In Figure 3b, there is the color distribution interspersed with curve of photopic vision. There are also points of maximum eye sensitivity for photopic and scotopic vision as shown in Figure 1 [21].

As can be seen in Figure 3a, various colored LEDs have various current-voltage curve steepness characteristics and consequently various behaviors, e.g., color instability of emitted light. In the forward direction, the stability of emitted light may be resolved with constant current control. It is possible to achieve the constant luminous flux by this control, which does not depend on ambient temperature, or circuit supply voltage.

**Figure 4:**
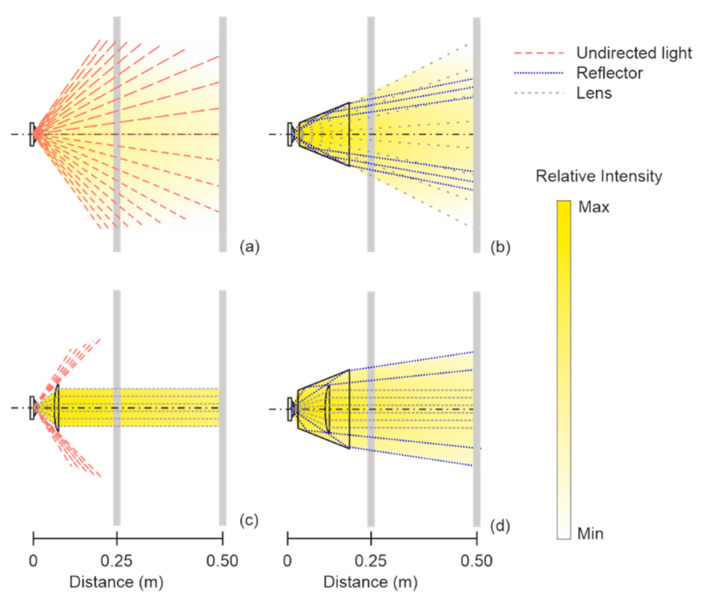
Operation radius of an LED (**a**) without a reflector; (**b**) with a reflector; (**c**) with a lens; (**d**) with TIR.

The use of reflector is an alternative possibility for increasing total efficiency of the light source which allows optimization to reach the maximum intensities. LED light sources emit the light into the one hemisphere with the spatial angles lower than 180 degrees in Figure 4a. LEDs radiate 100% of relative luminous intensity and by the higher spatial angle in its central axis, and from its central axis, the relative luminous intensity decreases. The total efficiency of light radiation can be increased with the directing of the light beam. The directing of light beam principle using a reflector modifies the radiation pattern, as shown in Figure 4b. Dimensions and the pattern of reflector can affect total luminous flux. Reflectors are also used for LED light optimization, especially COB (chip-on board) type LEDs that are designed as a LED field. The resulting behavior of the COB LED system depends on the quality of used reflector. Luminous flux losses can occur if the central beams are not efficaciously reflected and light is radiated outside of the desired direction.

The next possibility of system optimization and efficiency increase is the use of the optical lens in Figure 4c which allows the optimization of luminous flux, similarly to reflectors, by modification of the LED radiation characteristics. Similar to luminous flux modification is a TIR (total internal reflection), which combines the advantages of reflectors and lens used to reach for maximum efficiency in Figure 4d. The central beams are directed by primary and secondary lens and the reflector directs other beams. The lens principle is convenient for applications requiring a narrow and high intensity beam with small requirements to power consumption [22,23,24,25,26,27].

Light direction with reflector is the best option for the ASLODE application considering lens design attenuation and maintenance. The surface of the reflector must contain high-reflective material for light efficiency increase. The reflectors can reach reflectivity up to 97% when using high-purity aluminum.

By the design and realization, it is necessary not to cause permanent eyesight damage, i.e., the light intensity of perceived light must be under the level causing such permanent eyesight damage. These levels are defined in safety and hygiene standards that describe the rules for safe use of sources of bright light.

For the design of safe illumination, it is necessary to accept the ANSI Z136.1-2000 standard, which determines the *maximum permissible exposure* (MPE). Hygiene standards are built for the worst conditions of use — the 8 mm pupil diameter and eyesight adapted to scotopic (night) vision [28].

The threshold of eyesight damage is given by the upper limit of light intensity; which means radiant flux areal density is safe for human eyesight that reaches 0.0583 W/cm^2^ for extremely short mono-pulse exposure to 0.0001 W/cm^2^ and less for lengthened exposure. The value of 0.0001 W/cm^2^ is also considered as the lowest intensity limit for useful glare in flash lighting. Various levels of exposure have various types of psychophysiological effects, which depend on the exposure intensity and time. The basic types of negative effects to human eyesight are the glare, afterimages, and physiological disorientation—it is up to MPE limit. MPE 0.0026 W/cm^2^ is by the 0.25 s of exposure [28].

The MPE dose does not objectively express the level of the retina radiation exposure of the eyesight. The MPE value is defined for the eye with the maximum pupil diameter in scotopic vision. Besides considering the power of the source, the size of the irradiated area, and the spectral distribution of the intensity of the source, it is also necessary to incorporate the pupil diameter, accommodation, abstraction state, and distance when determining the degree of damage.

The subjective feeling of perceiving the observed brightness of the light source depends on the length of exposure. At exposures longer than 0.5 s, the perception of brightness decreases due to eye accommodation. Human vision is affected by the use of a stroboscopic light source such as the perception of flashing light. Human eyesight perceives light pulses as a continuous signal, such that the blinking period is proportional to the inertia of vision. The threshold frequency, or critical frequency of flash fusion, is the frequency at which a stroboscopic light source appears as a continuous light. The value of critical frequency is from 14 to 70 Hz, depending on the duty cycle, the shape, brightness, background, angular dimensions of the object, projection on the retina, the level of accommodation of eyesight, etc. [10,15].

The critical frequency of flash fusion is increased:

- If the signal brightness increases according to the Equation (6), e.g., the brightness change from 1to 120 cd/m^2^ causes the *F_Kr_* change from 14 to 35 Hertz. The critical frequency is given by equation:
  (6) $$F_{Kr}=a\cdot \log B+b,$$
  where *F_Kr_* is the critical frequency, *B* is the subjective feeling of perceiving the intensity of the flashing light, and *a*, *b* are the constants that depend on color.
- If angular dimensions of the bright object increases, e.g., a 5′ to 4°45′ change causes the *F_Kr_* to change from 14 to 44 Hertz; and
- With the change of duty cycle.

The subjective feeling of perceiving the intensity of the flashing light and the *F_Kr_* limit is the same as if the intensity of the flashing light was distributed throughout the exposure according to Talbot’s law [10,15]:

(7) $$B=\frac{L_{F}*\tau_{OFF}+L_{ASLODE}*\tau_{ON}}{\tau_{OFF}+\tau_{ON}},$$

where *L_F_* is the brightness of background, *L_ASLODE_* is the brightness of observed object, *τ_ON_* is the time of active shining, and *τ_OFF_* is the time without shining.

**Figure 5:**
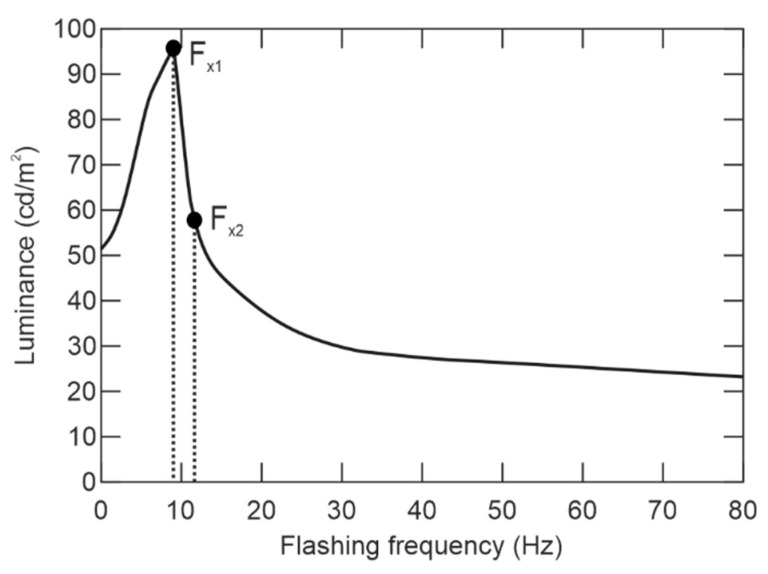
Subjective feeling of perceiving the brightness (adapted from [17]).

Figure 5 shows the subjective feeling of perceiving the brightness depending on flashing frequency, with the 50% duty cycle. It is evident that human eyesight is the most sensitive to the frequency *F*_×1_, however, from the perspective of eyesight protection, the *F*_×2_ frequency was chosen, where human eyesight is still sensitive enough.

**Figure 6:**
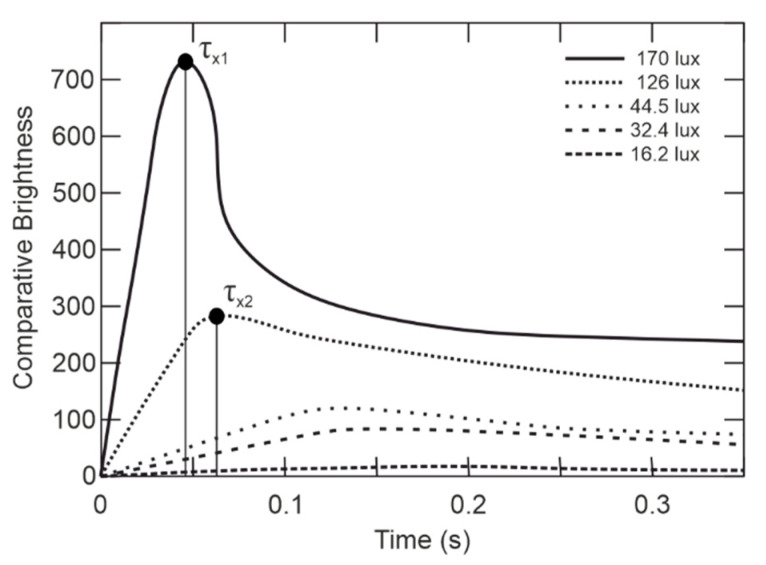
Apparent brightness of flashes with various luminances, as a function of flash duration (adapted from [3]).

Besides *F_Kr_*, the Broca–Sulzer effect influences perception if light is flashing, showing percept brightness is higher with the flashing beginning than continuous flashing. Apparent brightness of flashes and its dependence on pulse-width with various light intensities is shown in Figure 6. Figure 6 shows that the time duration of the pulse directly influences the subjective feeling of perceiving the brightness. In addition, the subjective feeling is not constant in various lighting conditions and changes with illumination. It means, the maximum comparative brightness *τ*_×1_ is circa 0.04 s of pulse duration in condition of 170 lx background, while on the other hand, *τ*_×2_ is circa for 0.04 s in 126 lx background.

The highlighted points in Figure 6 show *F*_×1_ and *F*_×2_ showing the major influence of eyesight sensitivity to the lighting mode. The human eyesight is most sensitive at *F*_×1_, where the subjective feeling of perceiving the brightness depends also on the pulse width, see Figure 6, color, intensity, etc. Using feedback from the smart-illumination sensor unit can adjust the optimal frequency and pulse width to reach the reliably glare effect over the various lighting characteristics on board the aircraft through the flight. The understanding of the human-eyesight determines the ASLODE design.

## 4. ASLODE Design

### 4.1. System Design

The basic principle of the designed device is the current control of every LED chip independently with a microcontroller. As for theimplementation, the RGBW LED light source is selected. Considering the design limits, two channels are implemented, the Y-channel (Red-Green) and W-channel (Blue-White). Every channel has its own exciter, driven by a microcontroller with feedback from the sensor-illumination unit. The microcontroller evaluates the press of the switch and accordingly sets the mode between normal lighting and attacking mode. In the normal lighting mode, only the W-channel is active, which is formed with white and blue LED chips and the final light emitted has a bit bluish color. In the attacking mode, both the Y and W-channels are switched affording full power. For the increase of glare effect, considering the accommodation of human eyesight; the frequency of flashes is 11 Hz in attack mode as shown in Figure 5, where the maximum effect to subjective feeling of perceiving the brightness is slightly under the flashing frequency of 10 Hz. The use of 11 Hz of flashing frequency takes into account the safety of the device and it is closely connected to hygienic standards as can be seen in paragraph 5.3. and Table 1.

### 4.2. Light Source Selection

The requirements of the light source for the glare equipment have tradeoffs: small physical dimensions, high light power with green color if possible, possibility of normal lighting, and operating by common batteries. Considering the requirements, a laser or LED light source can be used. As laser light source could have been the most suitable; nevertheless, there is a high risk of eyesight damage. The second disadvantage is the fact that laser is the source of monochrome light; and consequently, another light source should be used for normal lighting. It follows that the most applicable light source is the high luminous efficacy LED.

There are many options to design such a high luminous efficacy LED device. The first possibility is to select green LED as a source for glare and then add white light to the device. However, such an option is not convenient because of design possibilities. Therefore, the RGB/RGBW LED option was chosen. According to the technical data, the most suitable LED light source is LZ4-20MD00-0000 high luminous LED. It is 10 Watts RGBW LED. The chip consists of one red, one green, one blue, and one white LED chips. The whole chip is equipped with primary lens. The dimension of the chip is 7 × 7 mm. The MCPCB (metal core printed circuit board) option of the LED chip is most suitable for the defensive equipment.

Figure 3b shows the spectral distribution of individual LEDs on the chip, at 25 °C, according to the producer of the LED. The dominant wavelengths of individual LEDs are shown. The spectrum of white LED is given by phosphor material with high level of blue light. The relative intensity of blue in white light gives the final temperature of the light. It is visible from the spectrum, where the white LED displays a cold white color. Color correcting and changes depend on the temperature which can be corrected by a LED chip smart thermal sensor unit temperature measurement.

The dominant wavelength of the green LED is 525–530 nm. In fact, human eyesight is able to receive about 80–90% of LED relative light power. It is appropriate to use white color to amplify the green channel because white light contains the green color. For better glare effect, all colors in the area of photopic vision, which are available on the chip, can be used for additional intensification.

### 4.3. Driver Design and Illuminance Control

**Figure 7:**
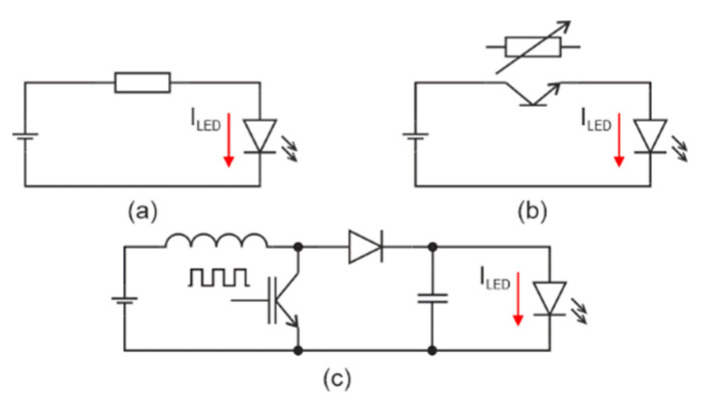
Basic principle of luminous flux drive (**a**) with resistor; (**b**) with transistor; and (**c**) DC/DC convertor (adapted from [5,33]).

The value of the current (*I_LED_*) has the effect on radiation intensity of the used LED which means that the luminous flux emitted by LED can be controlled by the current (*I_LED_*). Generally, there are several principles of current regulation that are incorporated into ASLODE from which the current is based on the smart-illumination unit measurements. With a ballast resistor in Figure 7a, a transistor in Figure 7b, or a SMPS (switch-mode power supplies) in Figure 7c; the final LED light intensity can also be controlled by PWM (pulse width modulation) by means of a shift. PWM does not control the current, but controls the transistor switching [29,30,31,32,33].

Considering battery applications, current control with ballast resistor is not suitable, as the LED current decreases with the discharging of the battery. In the LED current control with a transistor, it is possible to compensate the drop of input voltage. The total efficiency of LED current control with ballast resistor or transistor depends on the ratio of input voltage and LED working voltage.

SMPS (switch-mode power supplies) are universal tools to adapt the load to supply the source. These sources are designed as the constant current or constant voltage sources according to the power load characteristics. It is convenient to design SMPS as the constant current sources for LED load. In the case that the input voltage is higher than output voltage, a BUCK-type (step-down converter) SMPS is appropriate, and in the case that the input voltage is less than output voltage, a BOOST-type (step-up converter) SMPS is used. In addition, if the input voltage is less, the same or higher BUCK-BOOST-type SMPS can be used [33,34,35,36,37,38,39,40,41,42].

**Figure 8:**
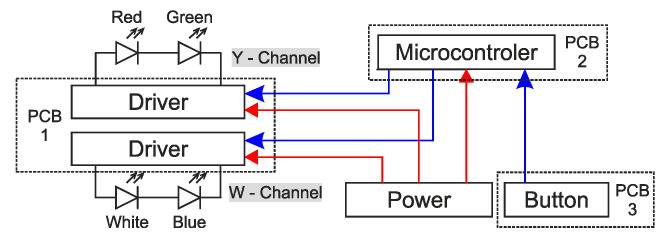
Block diagram of electronics in defensive device.

As for optimization of the number of components and printed circuit board (PCB) diameter reduction, it is not appropriate to control the LED chips independently, but merge them into the channels. The mode “flash light” is required too and therefore the channels are divided in the Y-channel consisting of red and green LED chips, and W-channel containing white and blue chips. The topology of electronics in designed defensive equipment shows the block diagram is illustrated in Figure 8.

The operating voltage increases with the serial connection of LED chips into the channels. Therefore, it is given that the driver must be BOOST-type. Considering the characteristics of supply and LED design, a BOOST-type LM3410 driver was chosen. The basic electrical characteristics of the driver are the working voltage from 2.7 V, maximum switched current 2.7 A, and switching frequency 1.6 MHz. Moreover, the integrated switching NMOS transistor provides illuminance control with the PWM signal. Because of high frequency of switching, a lesser value of the inductor and capacitor can be used.

**Figure 9:**
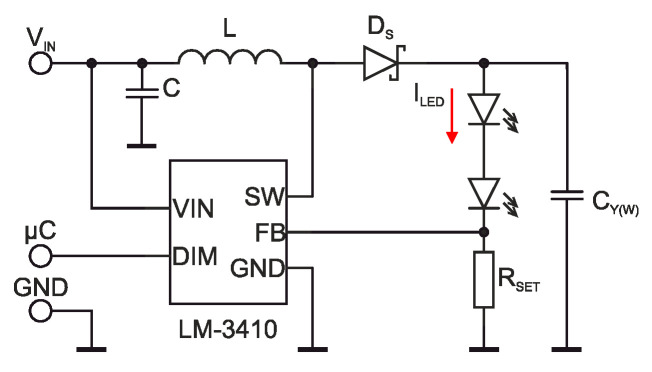
LED driver schematics (adapted from [43]).

There is the schematic diagram of LED driver for one channel in Figure 9. The following calculations belong to individual components [35,43].

The LM3410 driver contains in-built feedback for the LED current control. It reads the voltage on resistor *R_SET_*, where the driver keeps 190 mV. Considering the LED used and its electric characteristics, the required LED current is 0.9 A.

When calculating the supply unit, we choose the input voltage *V_IN_* according to the nominal voltage of the source, i.e., 4.5 V. The output voltage *V_OUT_* is chosen by the voltage on the individual LEDs determined by the current-voltage characteristics in Figure 3a at 0.9 A plus a *V_FB_* voltage by expression (8) for Y-channel and expression (9) for W-channel in equations:

(8) $$V_{OUT_{Y}}=V_{R}+V_{G}+V_{FB}=2.4+4.4+0.19=6.99\;V,$$

(9) $$V_{OUT_{W}}=V_{B}+V_{w}+V_{FB}=3.5+3.5+0.19=7.19\;V.$$

The driver regulates the forward current of the LED with keeping a feedback voltage on the reading resistor. The reading voltage is set by the manufacturer to 190 mV, and with selection of the reading resistor, the forward current is set according to Equation (10) [37,40,43]. The determination of resistivity for the current setting is according to the equation:

(10) $$I_{LED}=\frac{V_{FB}}{R_{SET}},$$

where *I_LED_* is the forward current of LED, *V_FB_* is the feedback voltage, and *R_SET_* is the resistivity of the reading resistor. After the substitution into Equation (10), the value is:

(11) $$R_{SET}=\frac{V_{FB}}{I_{LED}}=\frac{0.19}{0.9}=0.21\;\Omega .$$

The closest value for the reading resistor is 0.2 Ω and then the LED current is set on the current level 0.95 A. Due to PWM illuminance control (dual in-line memory module—DIMM pin), the illuminance can be a programmable set.

The inductor choice determines an output current ripple. The inductor current ripple is given by Equation (12). The current ripple can be in the interval 20–50% of the maximum forward LED current for the constant current source design. With maximum forward LED current *I_LED_* 0.95 A, determined by *R_SET_*, the inductor current ripple is 20%, or Δ*i_L_* 0.19 A. The inductor current ripple is given:

(12) $$\Delta i_{L}=\frac{V_{IN}}{2L}\cdot D\cdot T_{S},$$

where Δ*i_L_* is the inductor current ripple, *V_IN_* is the supply voltage, *L* is the inductance, *D* is the duty cycle, and *T_S_* is the switching period [37,38].

With a higher value of inductance, the current ripple is less but with bigger physical dimensions of the circuit. The shift determination, *D*, is given by input to output ratio [9], where *η* is the efficiency ratio as:

(13) $$D_{MAX}=\frac{V_{OUT}-\eta \cdot V_{IN}}{V_{IN}},$$

where *D_MAX_* is maximum duty cycle, *V_IN_* is supply voltage, and *η* is efficiency.

The duty cycle for the BOOST-type converter is approximated according to:

(14) $$D=\frac{V_{OUT}-V_{IN}}{V_{OUT}}.$$

To refine the calculations, it is appropriate to take into account the power losses caused by the Schottky diode, the integrated NMOS (N-type metal-oxide-semiconductor) switching transistor and the losses on the inductor given by (15). The efficiency is expressed by:

(15) $$\eta =\left(\frac{1-\frac{(1-D)\cdot V_{D}}{V_{IN}}}{1+\frac{R_{DCR}+(D\cdot R_{DSON})}{{\left(1-D\right)}^{2}\cdot R_{OUT}}}\right),$$

where *D* is the duty cycle, *V_D_* is the forward drop voltage of the Schottky diode, *R_DCR_* is the inductor series resistance, *R_DSON_* is the switch on resistance of the integrated NMOS transistor, and *R_OUT_* is the load resistivity [37,38,39,40,41].

In our case, to determine the maximum duty cycle, it is appropriate to use Equation (13) and set the efficiency *η* to 0.9 for both the Y-channel and W-channel. After the substitution, the values are:

(16) $$D_{MAX_{Y}}=\frac{V_{OUT}-\eta \cdot V_{IN}}{V_{IN}}=\frac{6.99-0.9\cdot 4.5}{4.5}=0.65\;(-),$$

(17) $$D_{MAX_{Y}}=\frac{V_{OUT}-\eta \cdot V_{IN}}{V_{IN}}=\frac{7.19-0.9\cdot 4.5}{4.5}=0.70\;(-).$$

With adjusting Equation (12) of the ripple, Equation (18) discerns the inductance for the inductor for the Y and W-channels, according to:

(18) $$L=\frac{V_{IN}\cdot D_{MAX}}{2\cdot \Delta i_{L}\cdot f},$$

where *f* is the driver switching frequency.

(19) $$L_{Y}=\frac{V_{IN}\cdot D_{MAX_{Y}}}{2\cdot \Delta i_{L}\cdot f}=\frac{4.5\cdot 0.65}{2\cdot 0.19\cdot 1.6\cdot 10^{3}}\approx 4.81\;\mu H,$$

(20) $$L_{W}=\frac{V_{IN}\cdot D_{MAX_{W}}}{2\cdot \Delta i_{L}\cdot f}=\frac{4.5\cdot 0.70}{2\cdot 0.19\cdot 1.6\cdot 10^{3}}\approx 5.18\;\mu H.$$

Considering 20% as the value tolerance of real inductors, 5.6 H inductors were chosen for both channels. To decrease losses, ferrite-core inductors are more suitable. The inductor saturation current must be higher than the sum of input current and current ripple.

As for input and output capacitors, the producer recommends using MLCCs (multilayer ceramic capacitors) technology. The recommended value of input capacitor should be chosen in the 2–22 µF interval and output capacitor of:

(21) $$C_{Y(W)}=\frac{V_{OUT}\cdot D_{MAX}}{2\cdot f\cdot R_{D}\cdot V_{OUT}},$$

where *C* is found capacity, *D_MAX_* is the maximum duty cycle, *f* driver switching frequency, *R_D_* is LED resistance, and *V_OUT_* is output voltage. 

For individual channels, the capacities are given by equations:

(22) $$C_{Y}=\frac{V_{OUT_{Y}}\cdot D_{MAX_{Y}}}{2\cdot f\cdot R_{D_{Y}}\cdot V_{OUT_{Y}}}=\frac{6.99\cdot 0.65}{2\cdot 1.6\cdot 10^{6}\cdot 7.36\cdot 6.99}\approx 27.6\;\mathrm{nF},$$

(23) $$C_{W}=\frac{V_{OUT_{W}}\cdot D_{MAX_{W}}}{2\cdot f\cdot R_{D_{W}}\cdot V_{OUT_{W}}}=\frac{7.19\cdot 0.7}{2\cdot 1.6\cdot 10^{6}\cdot 7.57\cdot 7.19}\approx 28.9\;\mathrm{nF}.$$

### 4.4. Electronic Control and Sensor Unit

ATTiny85 [13] in package SOIC.8 was chosen for control. The ATTiny85 microcontroller works with an integrated oscillator from supply voltage of 1.8 V. It contains six input/output pins, two that support PWM. The processor supports sleep mode with consumption of 0.1 µA by 1.8 V. An external oscillator can be added to microcontroller, where the working frequency and its stability can be increased based on the sensed conditions. The working frequency and stability of internal oscillator is sufficient for the ASLODE application [44].

With the connection to the supply unit, the initialization is made with the input/output port settings, and the code waits 2 s after a button press. If the button is not pressed, the microcontroller goes into sleep mode. Almost all the microprocessor functions are deactivated in sleep mode to lower the consumption to the level of 10^0^ µA. With the press of button, the microprocessor goes to waiting mode and activates all the functions.

**Figure 10:**
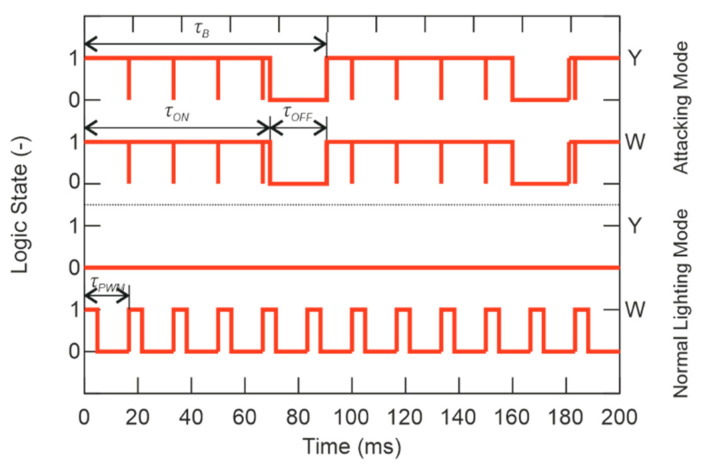
Channels timing.

The main code of the microcontroller follows from the press of the button. If the button is pressed, the main code for attacking mode runs. In attacking mode, both channels (W and Y) are switched on with full light intensity and 11 Hz flashes. As mentioned before, see Figure 5 where F_×1_ is presented as the switching frequency, where the human eyesight is the most sensitive. Otherwise, as for safety of eyesight, where it can be damaged very easily, the F_×2_ = 11 Hz of flashing frequency was chosen. The principle of control of ASLODE can be seen in Figure 10.

The control code is set so that the flashing frequency, flashing shift, and light intensity are the same for the Y-channel and the W-channel. It is assumed from a series of laboratory tests, that the flash parameters can be changed to increase the device’s efficiency to humans while reducing the risk of permanent damage to the human eyesight.

The channel timing of individual channels (Y-channel and W-channel) in Figure 10, depends on the mode of operation. The PWM signal with *τ_PWM_* period and frequency of 60 Hz is the main signal. By duty cycle change, the output signal changes the output LED current *I_LED_*, expressed by:

(24) $$I_{LED}=D_{PWM}\cdot I_{LED(\max)},$$

where *I_LED_* is the LED current, *D_PWM_* is the duty cycle of the PWM signal, and *I*_*LED*(max)_ is the maximum current for which the driver is rated, see Equation (10).

**Figure 11:**
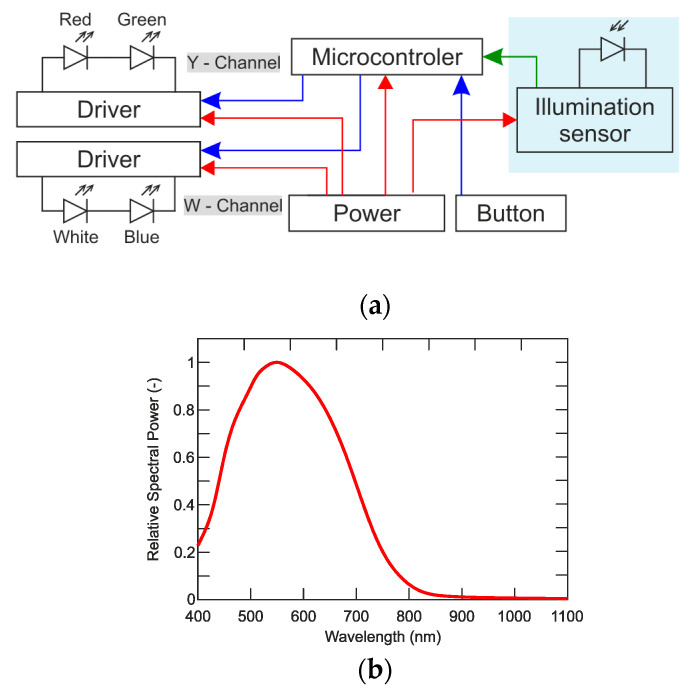
(**a**) The ASLODE block diagram with smart-illumination sensor; (**b**) relative spectral intensity of sensor (adapted from [45]).

As shown in Figure 10, the PWM signal is used in the attacking mode, where the maximum current of the LED is determined by the smart-illumination sensor unit feedback, without the need of intervention in the printed circuit board (PCB), or the need to change the timing of flashes *τ_B_*. The period *τ_B_* and the ratio *τ_ON_* to *τ_OFF_* affect the critical frequency *F_Kr_* as shown in Figure 6. The subjective feeling of perceived brightness is given not only by the flashing frequency, but also by the duration of one flash *τ_ON_*. In the normal light mode, the Y-channel is disconnected and only the W-channel with 30% *D_PWM_* of duty cycle, which means that the device’s light intensity in the normal lighting mode is only 30% of the maximum W-channel’s light intensity. Eyesight is still sensitive enough to keep the maximum of efficiency; however, this frequency is combined with the convenient amount of light as well as the colors used to maintain the maximum safety. ASLODE adjusts the constant flashing frequency and pulse width based on the smart illumination sensor unit. In case of efficiency optimization, it is necessary to include the lighting conditions of the attacker’s eyesight, and coincidentally improve the total efficiency. In Figure 11a, there is the optimized block diagram of defensive device, the sensoric part is added, which influences the settings of control pulses with the current lighting conditions [30].

To evaluate the illumination, a sensor whose sensitivity corresponds to the sensitivity of human eyesight can be used. The photosensor uses a photosensitive material to change electrical characteristics. The most common sensors include photoresistors, phototransistors, and photodiodes. When choosing the photosensor, the sensitivity of the sensor must be taken into account so that its spectral sensitivity is as close as possible to the spectral sensitivity of the human eyesight. Figure 11b shows the relative spectral sensitivity of the high-speed PIN photodiode [45] with 550 nm of the maximum spectral sensitivity, which ensures that the sensitivity of the sensor to illumination almost corresponds to the sensitivity of the human eyesight. The control system is able to better evaluate current lighting conditions and consequently, the suitably set flashing parameters such as flashing frequency, flashing alternation, and flash intensity, as well as color of flashes.

The sensor can be placed on the body of the ASLODE device not to be shaded during use. The second option is to equip the device with low power wireless technology, e.g., Bluetooth LE, ZigBee, obtaining the lighting data from an external sensor. The practical implementation with the sensor is the subject of further development and research in cooperation with the users of this device. The aim of the project is to solve the physical location of the sensor and test the flash modes for determined lighting levels.

One of the basic requirements was not to activate the attack lighting mode instead of the normal mode. From a structural point of view, it was not possible to use two buttons. Therefore, modes are controlled by only one microprocessor button press. It is necessary to perform a quick double-click to switching on the flashlight mode. By pressing the button again, the flashlight mode ends.

## 5. Practical Implementation and Experimental Verification

### 5.1. Implementation

Considering the available physical dimensions in design, the ASLODE device consists of the PCB with drivers and a microprocessor. The circuit is based on the producer’s recommendations and optimization for the desired application. The smart-illumination sensor unit is offline and determines the settings for ASLODE which can be wirelessly sent to the device.

**Figure 12:**
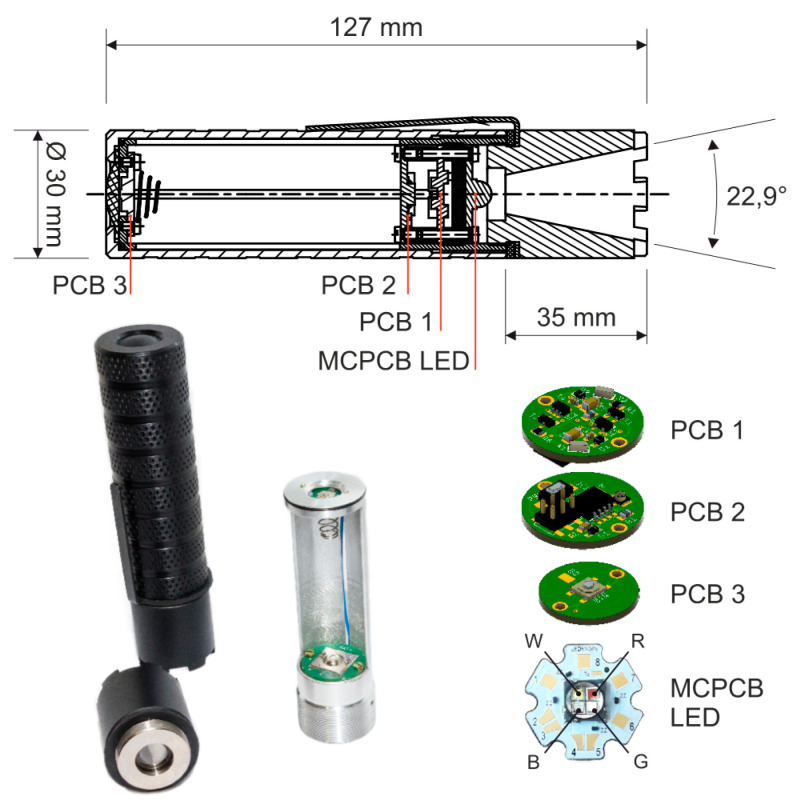
Implementation and printed circuit board design.

Figure 12 presents the design of a functional prototype. The ASLODE device contains its body carrying electronics and LED, cover and reflector. As seen in Figure 12, the batteries are not inserted directly to the body, but into the battery holder. The batteries can be inserted into the body as a module, with two advantages, simpler design and faster discharged battery exchange.

The body of the ASLODE device is equipped with anti-slip surface and with an optional clip for hanging, e.g., behind the belt. The basic tactic of use is the quick withdrawal from the protective case and the use in the clenched fist. The body can also be equipped with the strap to protect ASLODE from being pulled out of the hand.

As shown in Figure 12, the light directing is realized by LED primary lens and the reflector (the principle is shown in Figure 4b). The reflector use or total internal reflection (TIR) is more suitable as the design is simpler and the material reflectivity can be very high. The length of the reflector is 35 mm, and the beam angle is set at 22.9°. The light emitting spatial angle of this reflector is given by Equation (25) and the illuminated area at the distance of 0.5 m is given by Equation (26). The spatial angle is given by:

(25) $$\Omega =2\cdot \pi \cdot \left(1-\cos\left(\frac{\varphi }{2}\right)\right)=2\cdot 3.142\left(1-\cos\left(\frac{22.9}{2}\right)\right)=0.125\;\mathrm{sr},$$

where Ω is the spatial angle and *φ* is the planar beam angle of the reflector. The illuminated area is given:

(26) $$A_{P}=\Omega \cdot r^{2}=0.125\cdot 0.5^{2}=0.031\;m^{2},$$

where *A_P_* is the illuminated area, Ω is spatial angle, and *r* is the distance between the center of the source and the illuminated surface [5,6].

### 5.2. Experimental Verification

**Figure 13:**
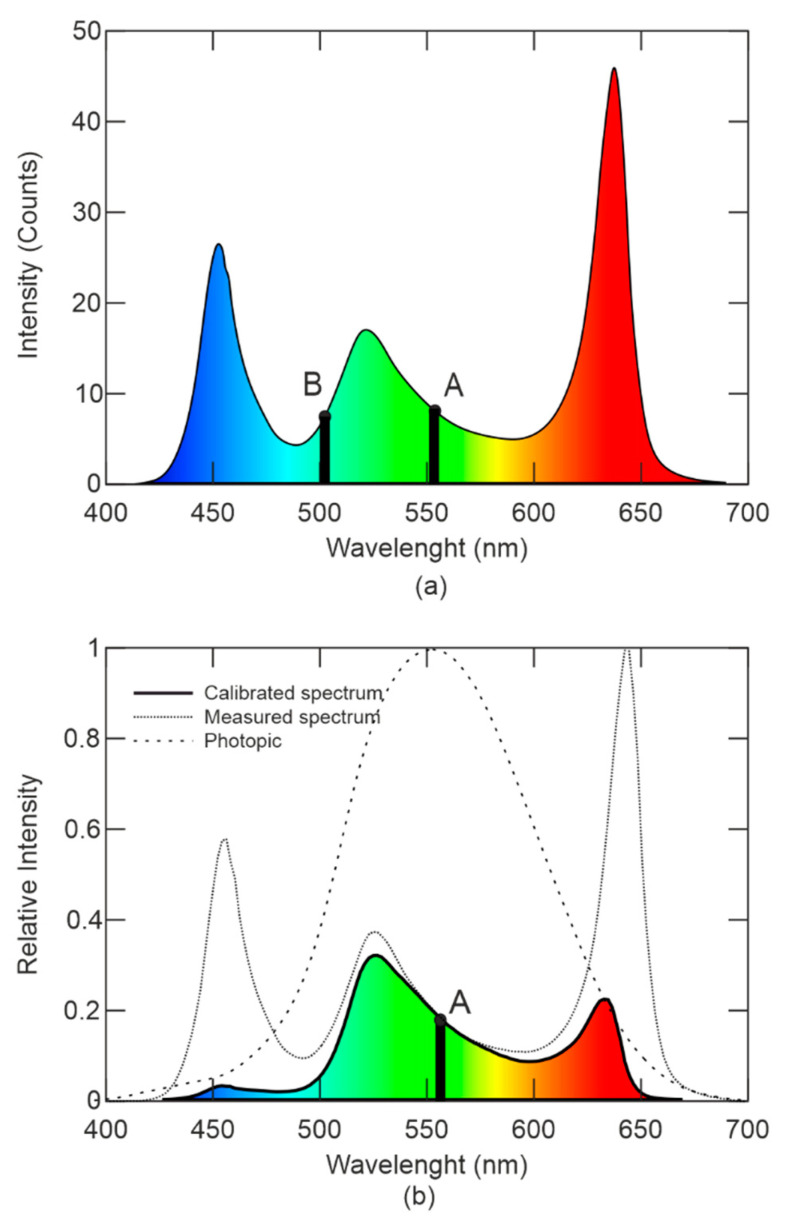
Y+W-channels: (**a**) emitted spectrum (**b**) calibrated spectrum for photopic vision.

The spectra of the Y and W-channels are measured to verification of the selected LED which are supplied with 950 mA of constant current. The spectrum is shown in the Figure 13a. The Y-channel spectrum contains a high amount of green color with the maximum wavelength of 520 nm and the red color with the maximum of 635 nm. The W-channel contains the blue color and it is visible that resulting light is the cold white with the high amount of blue component, although the W-channel also contains the significant amount of green color. Reaching an efficacious glare, both channels are activated in the attacking mode and the amount of green color is increased, where human eyesight is the most sensitive [46,47].

In Figure 13b, the calibrated spectrum is interleaved with the photopic area and the resulting relative light intensity perceived by the human eyesight of the selected LED. As seen in Figure 13b, although the selected LED parameters (based on safety considerations) have the relatively high light intensity in the blue and red areas, most of the light of the human eyesight is directly in the green area. Another consideration is the distance.

The luminous intensity decreases with the square of the distance. So, the resulting effect of defensive device depends on the sensed distance as well as others factors such as the amount of glare of the attacker and operational safety. Nevertheless, for very small distances, the light can easily reach the threshold limits (shown the Table 1), and consequently damage attackers’ eyesight. The threshold limits are set by very strict standards.

**Figure 14:**
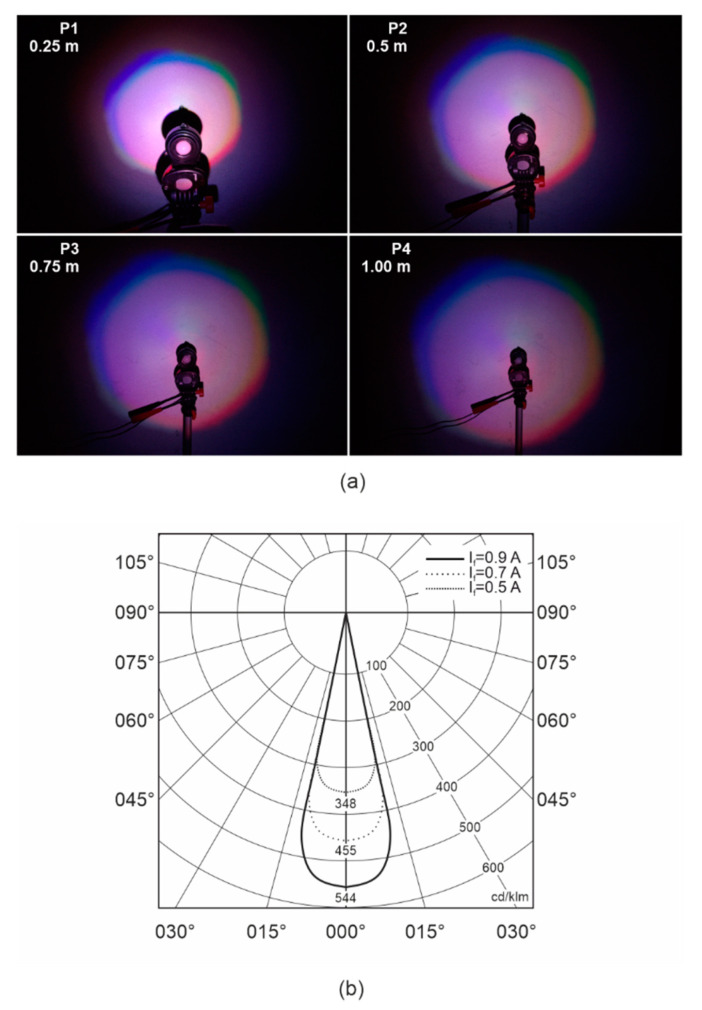
Verification of light intensity depending on the distance: (**a**) total light pattern; (**b**) luminous intensity distribution curve of ASLODE.

Figure 14a shows the total light pattern for distances of 0.25, 0.5, 0.75, and 1.00 m. Since the LED chips are not in one axis, but in the matrix around the center component patterns, the colors are slightly shifted. Consequently, every color has its illumination axis in its own position and thereby its own maximum. The correlated color temperature is 6288 K in the center of the light pattern featuring more light in the blue range. Figure 14b presents the luminous intensity distribution curve of ASLODE.

### 5.3. Onboard Aircraft Measurements

To verify the efficiency of the ASLODE defensive device on board the aircraft, the interior lighting of the Airbus A-319 aircraft was measured by the smart-illumination sensor unit (SISU) at LKKB (Prague–Kbely) Airport. In daytime conditions, the maximum outdoor illumination in the horizontal plane reaches 8950 lx.

**Figure 15:**
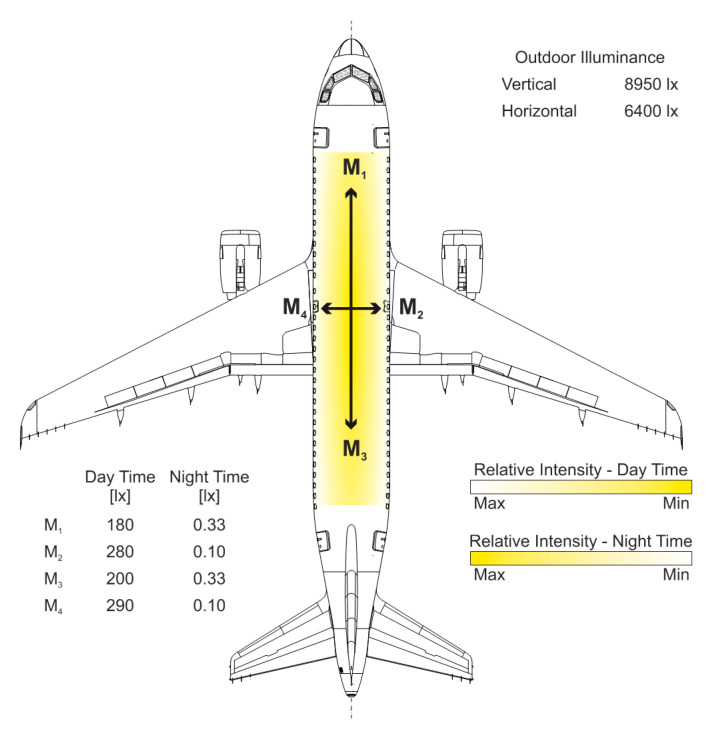
Results of measurement of illumination in the Airbus A-319CJ interior.

The SISU measurements took place in the front, middle, and rear of the aircraft fuselage, always in the axis of the aircraft fuselage with the sensor oriented into the front, rear, left, and right part according to Figure 15. The lighting level in the interior of the aircraft reaches 170–860 lx in the height of 170 cm above deck on the direction of the lux meter and with intensity of outdoor lighting. The level of interior lighting in dimmed windows is significantly affected by outdoor light, which depends on both the time of year and time of day, as well as factors like clouds.

**Figure 16:**
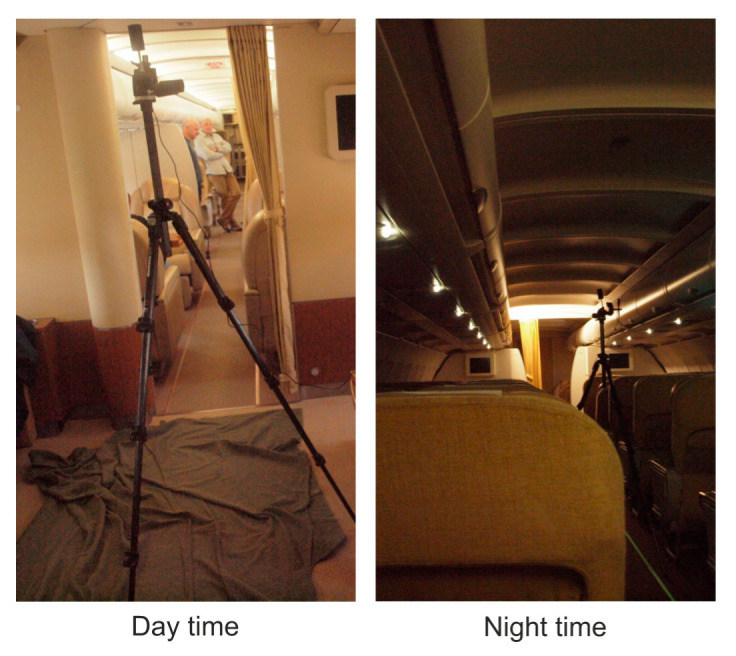
Measurement of illumination parameters to determine ASLODE on board the Airbus A-319CJ.

In the case of night mode, with covered windows and position lights on, the lighting intensity values are: M_1_ = 0.33 lx, M_2_ = 0.10 lx, M_3_ = 0.33 lx, and M_4_ = 0.10 lx. The photo from the measurement is shown in Figure 16.

As from the SISU measurement of the light intensity of the interior lighting of the aircraft board, the human eye can be in the vision areas of photopic (day conditions), mesopic (nighttime with interior lights), or scotopic vision (nighttime with interior lighting off). The fact that the conditions onboard the aircraft are not constant throughout the flight in day-time, year, and season, it is difficult to optimally control the SISU for effective operation from photopic to mesopic vision with respect to eyesight protection from damage.

The frequency of stroboscopic flashing of the defensive device is *f_B_* = 11.05 Hz, based on the lighting time within the one period being *τ_ON_* = 68.8 × 10^−3^ s and the dark phase *τ_ON_* = 21.7 × 10^−3^ s. In daytime conditions, on the board of the aircraft, the measured SISU luminance *L_d_* = 274 cd/m^2^ determines the luminance of the ASLODE defensive device with simultaneous emission of all four LEDs at the distance *r* = 0.5 m is *L_ASLODE_* = 158452 lm/(m^2^ sr) = 158,452 cd/m^2^. After substituting these values into Talbot’s law—Equation (7), the subjective feeling of brightness is *B_d_* = 120 (-). In daytime conditions on board the aircraft, the stroboscopic ASLODE defensive device appears approximately 120 times brighter than the continuously shining defensive device.

In the nighttime conditions onboard the aircraft, the SISU measured luminance is *L_n_* = 0.33 cd/m^2^ (mesopic). After substituting in Talbot’s law, Equation (7), *B_n_* = 120,458 (-). In the nighttime conditions onboard the aircraft, the stroboscopic defensive device appears approximately 120,000 times brighter than the continuously shining device. Further, circa 1000 times brighter than in daytime conditions. So, the use of the defensive device could have relatively high risk of permanent eyesight damage, which could be photochemical or thermal.

### 5.4. Verification of Safety for Human Eyesight

The determination of the effect on the human eyesight and critical exposure times must be based on hygienic standards and take into account the spectral distribution of the light source, exposure time, eye transmittance, and other parameters that will objectify the real effect on the human eyesight.

The determination of the safety of the device for the human eyesight is based on the European Union regulation on the minimum health and safety requirements regarding the exposure of workers to risks arising from physical agents. The biophysical effects of incoherent radiation depend on the wavelength of the optical radiation. The highest acceptable values are determined by integrals over the range of wavelengths weighted by spectral weighted coefficients [48]:

(27) $$L_{B}(t)=\int_{\lambda_{1}=300\;\mathrm{nm}}^{\lambda_{2}=700\;\mathrm{nm}}L_{\lambda }(\lambda ,t)\cdot B(\lambda )\cdot d\lambda ,$$

(28) $$L_{R}(t)=\int_{\lambda_{1}=300\;\mathrm{nm}}^{\lambda_{2}=700\;\mathrm{nm}}L_{\lambda }(\lambda ,t)\cdot R(\lambda )\cdot d\lambda ,$$

(29) $$E_{B}(t)=\int_{\lambda_{1}=300\;\mathrm{nm}}^{\lambda_{2}=700\;\mathrm{nm}}E_{\lambda }(\lambda ,t)\cdot B(\lambda )\cdot d\lambda ,$$

where *L_B_*(*t*) [W∙m^−2^∙sr^−1^] is the maximum spectral radiance for protection against photochemical damage, *L_R_*(*t*) [W∙m^−2^∙sr^−1^] is the maximum spectral radiance for protection against thermal damage. *L_λ_*(*λ,t*) [W∙m^−2^∙sr^−1^∙nm^−1^] is the spectral radiance, *E_λ_*(*λ,t*) [W∙m^−2^∙nm^−1^] is the spectral density of the luminous flux, *B*(*λ*)[–] is the spectral weighting coefficient taking into account the photochemical damage, and *R*(*λ*)[–] is a weighting coefficient taking into account the thermal damage of the eyesight.

To protect the eyesight from permanent damage with the visible and infrared spectrum radiation exposure from broadband sources in the interval λ ϵ [400–800], the source of such radiation must meet other criteria according to the characteristics presented in directive [48]. The main criterion is the sum of *B*(*m*) spectral emissions *L_λ_* of the source, weighted by the spectral hazard coefficient *B_λ_* of photochemical retinal damage, multiplied by the exposure time of the eyesight *τ_e_* [s], for the exposure time *τ_e_* <10^4^ must not exceed the limit value of 10^6^ [J∙m^−2^∙sr^−1^], by:

(30) $$\tau_{e}\cdot B(m)=\tau_{e}\cdot \sum_{400}^{800}L_{\lambda }\cdot B_{\lambda }\cdot \Delta \lambda \leq 10^{6}\;J\cdot m^{-2}\cdot sr^{-1}.$$

**Figure 17:**
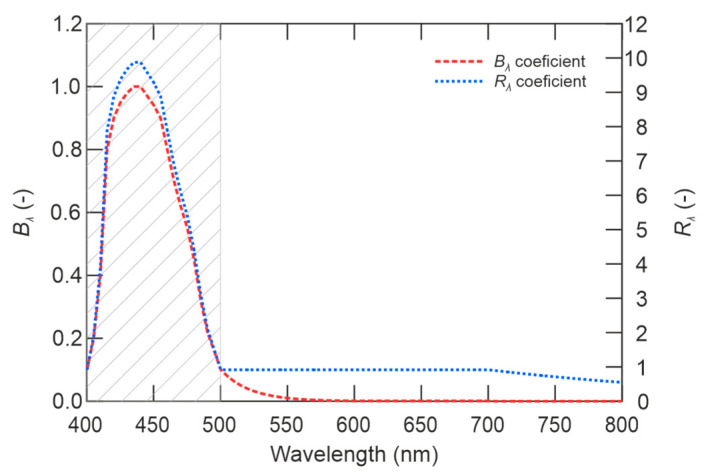
Spectral weighting coefficients *B*_λ_ and *R*_λ_ (adapted from [48]).

The value of the spectral radiance *L_λ_* is determined at the space of the eye of the exposed person. The values of the *B_λ_* and *R_λ_* coefficients are shown in the graph of the dependence of the coefficients on wavelength in Figure 17.

As can be seen in the Figure 17, in the grey area, there is the high risk of thermal and photochemical damage in the wavelength range [400–500] nm. This is the area of blue color light, the human eyesight is relatively less sensitive to glare; however, there is a high risk of eyesight damage. Furthermore, it is evident from the graph that the risk of thermal damage continues from the area of the visible spectrum to the area of IR radiation. The curve of *R_λ_* coefficient shows that the IR radiation still affects the eyesight by the thermal effect, which is why *R_λ_* has non-zero value in infrared wavelengths above 800 nm. When measuring the radiation of the selected LED, no radiation was detected in the IR spectrum, as shown in Figure 13a,b.

To protect the retina from the thermal damage, the exposure time *τ_e_* [s] must not be higher than *τ*_max_ according to:

(31) $$\tau_{\max}=\frac{K^{2}}{{\left(\alpha \cdot \sum_{400}^{800}L_{\lambda }\cdot R_{\lambda }\cdot \Delta \lambda \right)}^{2}},$$

where constant *K* = 104 [W∙m^−2^∙sr^−1^∙rad∙s^0.5^], *α* [rad] is the angle of view of the light source from the observer’s eye, *L_λ_* [W∙m^−2^∙sr^−1^] is the spectral radiance of the light source at the site of the exposed person’s eyesight, *R_λ_* is a weighting coefficient that takes into account the thermal damage of the eyesight, and Δ*λ* is the wavelength interval.

The selected LED light source LZ4-00MD00 emits incoherent light in the spectrum *λ*ϵ [400–800], the diameter of defensive device aperture is *D* = 30 mm, irradiated surface *A_p_* = 0.031 m^2^, and spatial angle of radiation is Ω = 0.125 sr. The flashing frequency of the device is set at 11.05 Hz with a duty cycle of 76%, which means that the exposure time in one period is 68.8 ms. The mean value of the protective response of the human eyesight is *τ_o_* = 0.25 s, which is the time taken for eyelid closure. By considering the protective time of the human eyesight and the SISU response, the maximum number of flashes is 3. Therefore, based on the average SISU measurements, the duration of exposure time in the maximum number of flashes is 0.206 s.

Table 1 shows the calculated thresholds values for eyesight damage for individual LED chips as well as for common emission based on the SISU measured conditions. The selected parameters are: exposure time *τ_e_* = 0.206 s, irradiated surface *A_p_* = 0.031 m^2^, spatial angle of radiation Ω = 0.125sr, and the distance of the light source from the eye *r* = 0.5 m.

The threshold values of human eyesight damage are given by expression (30) and expression (31) for photochemical damage.

In the flashing mode, it is shown from the calculated values, there is the risk of thermal damage to the eyesight with the exposure time longer than 7.96 s and photochemical damage with an exposure time longer than 1056 s. If the device is used at the distance of 0.1 m or less, permanent eyesight damage may occur.

Based on the proven analysis and measurements, it is shown that the flashing frequency of the final version of defensive equipment has a very wide range. Using feedback from the SISU, the control circuit optimizes the flash frequency according to the mode of the device as well as the primary use. Furthermore, as it is shown in Table 1, individual LED segment switching is optimized not to damage eyesight of the attacker—as the nature of defense mainly discontinues the attack, not to hurt the attacker and not cause injury to crew or other passengers. Table 1 shows that the blue light can cause eyesight damage in a very short time. From this, the flashing frequency should not have a long duration to limit eyesight damage by the thermal effect. As the blue W-channel use reaches the threshold level, there is the theoretical possibility the use of Y and W-channels in different ratios. Supposing a worst-case situation is when the SISU determines extreme darkness onboard the aircraft in the passenger zone, the pupil of the attacker opens the eye in the maximum diameter and the eyesight is the most sensitive. Thedevice must have efficient glare effect as well as the action of the device should be limited not to cause eyesight damage.

The blue color effect could be replaced by safer red-green Y-channel, which can be in action for a longer time to reach efficient glare effect. In Table 1, the duration of Y-channel action could be longer, or the W-channel might be completely unused; nevertheless, common batteries realize the supply of the device and such use would lead to fast discharge and consequently very limited use. At the same time, the red and green channel might have limited life. In addition, the psychological effect of blue color, in comparison to other colors, is very intensive and that is why the blue color must be used in such device to keep its efficiency. On the other hand, the use of blue color must take in consideration very strict hygienic standards [48], as the device should not damage the attacker’s eyesight. However, the settings of W and Y-channels can be very accurately controlled by the SISU/microprocessor, following all hygienic standards, securing the eyesight from thermal or photochemical overload.

The optimal setting of the microprocessor based on the measured data from the SISU enables intelligent adjustment so that the reaction time of the eyesight can be used to adjust the light intensity. However, the optimal setting is also related not only to the efficiency of the device, but also to its safety of operation onboard the aircraft. From the above analyzes, it is suitable to set the flash frequency around 8 Hz for environments with a high level of illumination, see Figure 5. For environments with low-light intensity, it is suitable to set the flash frequency higher than 8 Hz, up to 70 Hz. The SISU with a distance device can adjust the safe flash. Furthermore, the lengths of the pulses (flashes) are longer. SISU measured parameters are designed so that glare occurs on a clear day and at the same time, does not damage the eyesight at night. Such mode of the device is required, as it backs up the function, in case of sensor damage, or in case of its incorrect function.

The SISU provides for effective ASLODE parameter settings. From the presented analyzes of the effect of ASLODE on human eyesight, it is clear that the use of blue color at high intensities is dangerous and is limited from the safety point of view on board the aircraft by hygienic standards. Especially, the use of blue color in low-light conditions, see Figure 16b, where thermal damage to the retina may occur, the use of blue light must be limited or replaced by other components of the color spectrum with higher intensity. On the other hand, the components of blue light are very effective in glare. Thus, in the case of higher light intensities, where there may be a problem of realization of lighting, the blue color is very effective and also causes para-images which are required for the normal function of the defensive device. As shown in Figure 5, flashes with the frequency of about 9 Hz cause the brightest light due to the dynamic characteristics of the human eyesight, and if the light flashes above 30 Hz, the flashing merges into continuous illumination. Not only does the flashing frequency influence the subjective perception of brightness, but also doesthe pulse time *τ_ON_*, shown in Figure 6. The smart pulse setting method allows setting the flashing frequency from 8Hz for high level light intensities, up to 20 Hz in low level lighting conditions. Furthermore, there is the change in the length of the pulse and a change in color composition; especially the blue components are strictly controlled not to damage the eyesight of the crew, passenger, and attacker.

Future results and the ASLODE design would include active selection either by wireless transmission or by compact incorporation into ASLODE-II.

## 6. Conclusions

The article details the design of the incapacitating defensive aviation safe LED-based optical dazzler equipment (ASLODE) device utilizing a smart-illumination sensor unit (SISU) to judiciously choose the flash frequency for a measured lighting situation. Such an example is to repel a potential attack on an aircraft without an attacker or bystander injury, as the aggressor can be a member of the cabin crew or a passenger without primary intention to endanger the flight. A potential use of ASLODE is onboard an aircraft which has additional requirements, e.g., not to damage the aircraft fuselage or other aircraft systems, which requires a SISU for effective and efficient performance. The paper shows the approach to design such an incapacitating defensive device based on the light intensive glare. The dazzle glare disables a person’s eyesight; however, the emitted light cannot damage the eyesight. For the design, the human eyesight was taken as an adaptable sensor, where its adaptation parameters were the basis for the selection of the light source characteristics, lighting mode, as well as its emitted spectrum. The analysis of the human sensor and the SISU is required for ASLODE to balance device efficiency and the hygienic safety to determine the ASLODE emitted light for a strong enough and efficient glare effect; it is a function of the ambient lighting. The SISU provides a feedback loop to optimally set the illumination, flashing frequency, as well as color components of light to reach maximum glare, keeping the needed level of safety. The ASLODE design includes the controlled high luminous efficacy LED using the microprocessor system and the SISU.

Experimental measurements on an Airbus A 319CJ aircraft demonstrated the effectiveness of ASLODE to produce the flash for typical lighting conditions in common aircraft. Analysis and implementation show that the human eyesight can be effectively dazzled in typical situations and the ASLODE design secures effective and safe use onboard the aircraft in all typical lighting conditions that can occur during a typical passenger flight.

All calculations and efficiency analysis are based on the hygienic prerequisite standards that the device is used for a person without any eyesight defect and with the average protective measures (e.g., self-closing) for an eyesight reaction time of around 250 ms. Based on the average SISU measurements, the duration of exposure time for the maximum number of flashes of 3 is 206 ms for the ASLODE RGBW LED. In the case of a person with an eyesight defect, from which the person might use glasses, ASLODE’s efficiency could be reduced, or, conversely without glassware protection, ASLODE might cause serious consequence to the attacker’s eyesight.

## Figures and Tables

**Table 1:** Threshold limits of human eyesight damage.

	Thermal Damage (30)		Photochemical Damage (31)	
	$\sum L_{\lambda }\cdot R_{\lambda }\cdot \Delta \lambda$	$\tau_{e}\left[s\right]$	$\sum L_{\lambda }\cdot B_{\lambda }\cdot \Delta \lambda$	$\tau_{e}\cdot B\left(m\right)$
	[W∙m^−2^∙sr^−1^]	[s]	[J∙m^−2^∙sr^−1^∙s^−1^]	[J∙m^−2^∙sr^−1^]
R	5100	1067	5.21	1.07
G	3974	1758	137.8	28.22
B	49,400	11.38	4942	1018
W	537	96.327	43.75	9.012
RGBW	59,047	7.96	5128	1056

## Data Availability

Not applicable.

## References

[1] Liou H.-L., Brennan N.A. (1997). Anatomically accurate, finite model eye for optical modeling. J. Opt. Soc. Am. A.

[2] Davson H. (1990). Physiology of the Eye.

[3] Levin L.A., Kaufman P.L. (2011). Adler’s Physiology of the Eye: Clinical Application.

[4] Kim T., Lee E.C. (2020). Experimental Verification of Objective Visual Fatigue Measurement Based on Accurate Pupil Detection of Infrared Eye Image and Multi-Feature Analysis. Sensors.

[5] Lenk R., Lenk C. (2011). Practical Lighting Design with LEDs.

[6] Bukhshtab M.A. (2019). Photometry, Radiometry, and Measurements of Optical Losses.

[7] Maziarka J., Bena L., Wachta H. Meaning of Scotopic/Photopic Ratio of Light Sources in Lighting of Outdoor Spaces, 2018 VII. Proceedings of the Lighting Conference of the Visegrad Countries (Lumen V4).

[8] Standards and Technical Documents from International Commission on Illumination (1994). CIE 041-1978, Light as a True Visual Quantity: Principles of Measurement.

[9] Fan X., Yao G. (2011). Modeling Transient Pupillary Light Reflex Induced by a Short Light Flash. IEEE Trans. Biomed. Eng..

[10] Greene E. (2015). Evaluating Letter Recognition, Flicker Fusion, and the Talbot-Plateau Law using Microsecond-Duration Flashes. PLoS ONE.

[11] Björn L.O. (2015). Photobiology: The Science of Light and Life.

[12] Sokhn N., Wuilleret A., Caldara R. Go/No-Go Saccadic Reaction Times Towards Visual Field Targets Differ Between Athletes and Nonathletes. Proceedings of the 2019 11th International Conference on Knowledge and Smart Technology (KST).

[13] Gruzevič K.J. (2016). Optiko-Elektronnye Pribory Notchnogo Videnija.

[14] Bekkering H., Adam J.J., Huson A., Whiting H.T.A., Kingma H. (1994). Reaction time latencies of eye and hand movements in single- and dual-task conditions. Exp. Brain Res..

[15] Issolio L.A., Barraza J.F., Colombo E.M. (2006). Time course of brightness under transient glare condition. J. Opt. Soc. Am. A.

[16] Temporal Resolution by Michael Kalloniatis and Charles Luu. https://webvision.med.utah.edu/book/part-viii-psychophysics-of-vision/temporal-resolution/.

[17] Zhao S., Wang K., Chen F., Liu S. Optical design of LED packaging for concentrated and uniform lighting. Proceedings of the 2012 13th International Conference on Electronic Packaging Technology and High Density Packaging.

[18] Meschede D. (2017). Optics, Light, and Lasers: The Practical Approach to Modern Aspects of Photonics and Laser Physics.

[19] Ying S.P., Tang C., Huang B. Charaterizing LEDs for Mixture of Colored LED light sources. Proceedings of the 2006 International Conference on Electronic Materials and Packaging.

[20] Carreres-Prieto D., García J.T., Cerdán-Cartagena F., Suardiaz-Muro J. (2020). Performing Calibration of Transmittance by Single RGB-LED within the Visible Spectrum. Sensors.

[21] LED Engine (2018). High luminous efficacy RGBW LED emitter. LZ4-00MD00 Datasheet, November 2018.

[22] Xu Y., Chen G., Chang Y., Lin H., Zheng J., Gan L. The Secondary Optical Design and Fabrication for the Uniform Illuminating LED Spotlight Using TIR Lens. Proceedings of the International Seminar on Applied Physics, Optoelectronics and Photonics.

[23] Leung S.Y.Y., Zhong L., Wei J., Xu Z., Yuan C.C.A., Zhang G.Q. Optical design and characterization of micro-fabricated light reflector for 3D multi-chip LED module. Proceedings of the 2013 14th International Conference on Thermal, Mechanical and Multi-Physics Simulation and Experiments in Microelectronics and Microsystems (EuroSimE).

[24] Chen H., Wang S., Sun B., Guo H., Zhao W., Chang H., Zhang X., Liu H. Backside reflector using metallic mirror and ALD-TiO2/Al2O3 DBR for GaN-based LED. Proceedings of the 2012 IEEE 11th International Conference on Solid-State and Integrated Circuit Technology.

[25] Lui Q., Zhang Y., Zou N., Gao Y., Zhang J., Cao G., Cao G. Design of new hybrid optical system of LED cap lamp. Proceedings of the IET International Conference on Communication Technology and Application (ICCTA 2011).

[26] McCarthy A., Romero-Vivas J., O’Hara C., Rebrova N., Lewis L., Hegarty S.P. (2018). LED-Based Collimating Line-Light Combining Freeform and Fresnel Optics. IEEE Photonics J..

[27] Min K.-P., Kim J., Song K.D., Kim G.-W. (2019). A G-Fresnel Optical Device and Image Processing Based Miniature Spectrometer for Mechanoluminescence Sensor Applications. Sensors.

[28] American National Standards Institute (2018). ANSI Z136 Series, Laser Safety Standards.

[29] Lodi M., Oliveri A. (2020). Online Estimation of the Current Ripple on a Saturating Ferrite-Core Inductor in a Boost Converter. Sensors.

[30] Yan L., Yu Y., Hu S., Mulvaney D., Blanos P., Alharbi S., Hayes M. (2020). Illumination Adaptation in a Multi-Wavelength Opto-Electronic Patch Sensor. Sensors.

[31] Prudente M., Pfitscher L.L., Emmendoerfer G., Romaneli E.F., Gules R. (2008). Voltage Multiplier Cells Applied to Non-Isolated DC–DC Converters. IEEE Trans. Power Electron..

[32] Chen J., Prodic A., Erickson R.W., Maksimovic D. (2003). Predictive digital current programmed control. IEEE Trans. Power Electron..

[33] Forouzesh M., Siwakoti Y.P., Gorji S.A., Blaabjerg F., Lehman B. (2017). Step-Up DC–DC Converters: A Comprehensive Review of Voltage-Boosting Techniques, Topologies, and Applications. IEEE Trans. Power Electron..

[34] Lee P.-W., Lee Y.-S., Cheng D.K.W., Liu X.-C. (2000). Steady-state analysis of an interleaved boost converter with coupled inductors. IEEE Trans. Ind. Electron..

[35] Tseng K.C., Liang T.J. (2004). Novel high-efficiency step-up converter. IEE Proc. Electr. Power Appl..

[36] Leuchter J., Stekly V., Blasch E. (2015). Investigation of avionics power switch loading versus aircraft electromagnetic compatibility. IEEE Aerosp. Electron. Syst. Mag..

[37] Mohan N., Undeland T.M., Robbins W.P. (2003). Power Electronics: Converters, Applications, and Design.

[38] Leuchter J., Bauer P., Bojda P., Rerucha V. Bi-directional DC-DC converters for supercapacitor based energy buffer for electrical gen-sets. Proceedings of the 2007 European Conference on Power Electronics and Applications.

[39] Profumo F., Tenconi A., Cerchio M., Bojoi R. (2006). Fuel cells for Electric power generation: Peculiarities and dedicated solution for power electronic conditioning systems. EPE J..

[40] Luo F.L., Ye H. (2013). Advanced Dc/Dc Converters.

[41] Leuchter J., Boril J., Blasch E. Overview of Silicon Carbide Power Devices for Aircraft Electrical Systems. Proceedings of the 37thIEEE/AIAA Digital Avionics Systems Conference.

[42] Blasch E., Kostek P., Pačes P., Kramer K. (2015). Summary of avionics technologies. IEEE Aerosp. Electron. Syst. Mag..

[43] Texas Instruments (2007). Constant-current boost and SEPIC LED driver with internal compensation. LM3410 Datasheet, October 2007.

[44] Atlmel (2013). Atmel 8-bit AVR Microcontroller with 2/4/8K Bytes In-System Programmable Flash. ATtiny85 Datasheet, September 2013.

[45] Vishay (2017). Silicon PIN photodiode. VEMD5510C Datasheet, January 2017.

[46] Chang C., Chen C., Kurokawa U., Choi B.I. (2011). Accurate Sensing of LED Spectra via Low-Cost Spectrum Sensors. IEEE Sens. J..

[47] Stadler A. (2010). Analyzing UV/Vis/NIR Spectra—Correct and Efficient Parameter Extraction. IEEE Sens. J..

[48] The European Parliament and of the Council Directive (2006). 2006/25/ECThe Minimum Health and Safety Requirements Regarding the Exposure of Workers to Risks Arising from Physical Agents (Artificial Optical Radiation).

